# Synthesis of the Most Potent Isomer of μ-Conotoxin KIIIA Using Different Strategies

**DOI:** 10.3390/molecules28083377

**Published:** 2023-04-11

**Authors:** Xunxun Jian, Yong Wu, Zaoli Mei, Xiaopeng Zhu, Dongting Zhangsun, Sulan Luo

**Affiliations:** 1 School of Medicine, Guangxi University, Nanning 530004, China; janegut@163.com (X.J.); meizl1331@163.com (Z.M.); zhuxiaopeng@gxu.edu.cn (X.Z.); zhangsundt@163.com (D.Z.); 2 Key Laboratory of Tropical Biological Resources of Ministry of Education, Hainan University, Haikou 570228, China

**Keywords:** μ-conotoxins KIIIA, disulfide bond, oxidative folding, cysteine

## Abstract

In the chemical synthesis of conotoxins with multiple disulfide bonds, the oxidative folding process can result in diverse disulfide bond connectivities, which presents a challenge for determining the natural disulfide bond connectivities and leads to significant structural differences in the synthesized toxins. Here, we focus on KIIIA, a μ-conotoxin that has high potency in inhibiting Nav1.2 and Nav1.4. The non-natural connectivity pattern (C1—C9, C2—C15, C4—C16) of KIIIA exhibits the highest activity. In this study, we report an optimized Fmoc solid-phase synthesis of KIIIA using various strategies. Our results indicate that free random oxidation is the simplest method for peptides containing triple disulfide bonds, resulting in high yields and a simplified process. Alternatively, the semi-selective strategy utilizing Trt/Acm groups can also produce the ideal isomer, albeit with a lower yield. Furthermore, we performed distributed oxidation using three different protecting groups, optimizing their positions and cleavage order. Our results showed that prioritizing the cleavage of the Mob group over Acm may result in disulfide bond scrambling and the formation of new isomers. We also tested the activity of synthesized isomers on Nav1.4. These findings provide valuable guidance for the synthesis of multi-disulfide-bonded peptides in future studies.

## 1. Introduction

Conotoxins, derived from the venom of Conus venom, can interact with a variety of biological targets, such as transmembrane receptors, ion channels, and transporter proteins. This makes them an ideal molecular probe for studying specific isoforms of ion channels and receptors, as well as for drug candidate [1,2]. These conopeptides typically consist of roughly 10–50 amino acid residues and are rich in cysteines, which are essential for their biological activity and stability [3,4]. The M-superfamily of conotoxins is characterized by the CC-C-C-CC cysteine pattern and includes ψ-, μ-, and κM-conotoxins [3,5,6]. Among these, μ-conotoxins have been found to bind voltage-gated sodium channel pores and block sodium entry into cells [7,8,9].

Cysteine is an essential amino acid that plays a critical role in numerous biochemical processes, including oxidative folding, peptide structure stabilization, catalysis, metal binding, and cell signaling [10,11,12,13,14,15]. Disulfide bonds, formed between two cysteine residues, are crucial for the three-dimensional structure, biological activity, chemical composition, biophysical properties, and proteolytic stability of peptides and proteins [16,17,18,19,20,21,22,23]. Thus, the correct disulfide bonding is of utmost importance.

**Figure 1:**
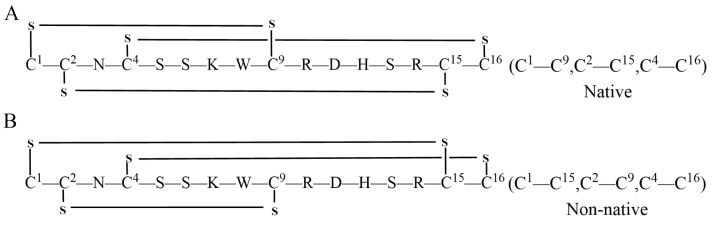
Two types of μ-KIIIA isomer, non-native isomer[C1—C9, C2—C15, C4—C16] inhibits Nav1.2 (**A**) and Nav1.4 (**B**) with high potency.

KIIIA is a 16-residue μ-conotoxin that has been proven to be the most efficient in blocking Nav1.2 and Nav1.4 in rats [24,25,26,27]. This conopeptide has different isomers, each with its own intradisulfide bond configuration (Figure 1). The disulfide connectivity of Cys1—Cys15, Cys2—Cys9, and Cys4—Cys16 is the most effective Nav1.2 inhibitor, possessing a dissociation constant Kd of approximately 5 nM [28] and significant potential for drug development. Previous studies have utilized various methods to synthesize different isomers of KIIIA [29,30,31,32,33]. The aim of this study is to synthesize a specific KIIIA isomer using different synthetic methods and to investigate the influence of the order of the protecting groups on the final product.

Synthesizing cysteine-rich peptides is a challenging task due to the formation of multiple disulfide bonds. Currently, three main strategies are employed, including free random oxidation, semi-selective formation, and stepwise regioselectivity. Although the free random oxidation method has been widely used, it requires further characterization of the disulfide bonding linkage. A semi-selective formation method is a hybrid approach that protects Cys residues with trityl (Trt)/acetamidomethyl (Acm) groups, resulting in three possible isomers. In contrast, stepwise regioselectivity involves carefully selecting protective groups for each Cys residue, and ideal isomers theoretically can be produced. Here, we selected three strategies to synthesize the most potent isomer of KIIIA (C1—C15, C2—C9, C4—C16). For stepwise regioselectivity, we further evaluated the effect of positions and cleavage order on the synthesis results. We also employed electrophysiological assays to detect the receptor activity of the synthesized isomers. Our results provide insights into the optimal strategy for synthesizing Cys-rich peptides and advance the development of drug candidates targeting ion channels and receptors.

## 2. Results

### 2.1. Chemical Synthesis of KIIIA with Free Random Oxidation Strategy

**Figure 2:**
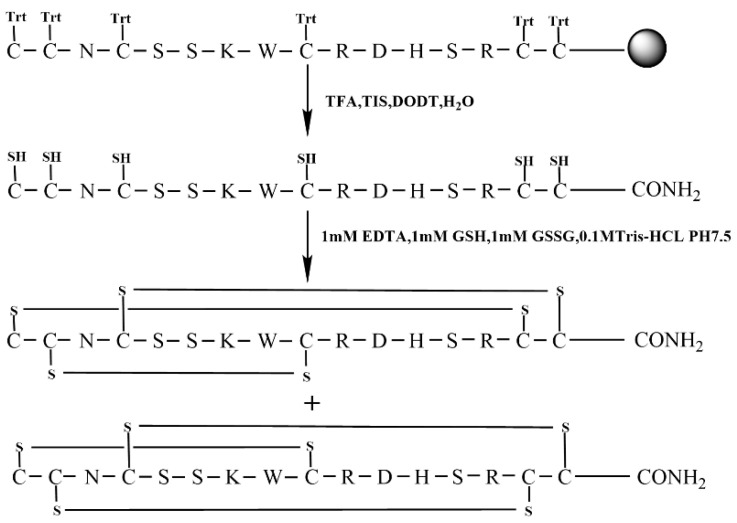
Free random oxidation strategy of KIIIA (Scheme 1).

The free random oxidation method is the simplest for the formation of disulfide bonds directly in a buffered aqueous environment. The specific synthesis line is shown in Figure 2. To begin, the linear KIIIA was synthesized with all six Cys residues protected by a trityl group.

**Figure 3:**
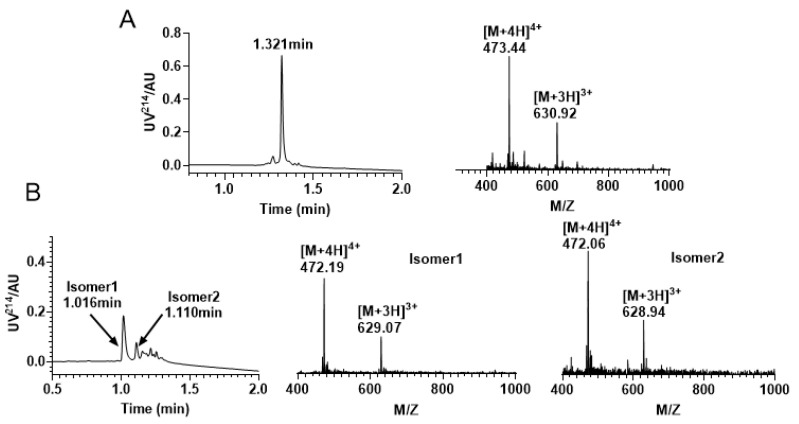
RP−UPLC and mass spectrometry traces of KIIIA by free random oxidation. (**A**) Linear RP−UPLC diagram of KIIIA with an observed mass of 1889.76 Da and a retention time of 1.321 min; (**B**) RP−UPLC diagram of the isomeric mixture produced by KIIIA, with an observed mass of isomer 1 1884.21 Da and a retention time of 1.016 min; The monitored mass of isomer 2 is 1883.82 Da and retention time is 1.110 min.

After successfully synthesizing the linear peptide (Figure 3A), we conducted oxidative folding in Tris-HCl buffer using a specific ratio of reducing and oxidizing glutathione. The process was closely monitored by RP−UPLC and ESI−MS (Figure 3B), which revealed the formation of two major isomers. The two main products can be separated from the mixture with the same molecular mass. Our target product is the isomer 1 (C1—C15, C2—C9, C4—C16), which can be distinguished from the product synthesized using the later directed strategy that co-eluted with it, which is consistent with the results of prior studies [30].

### 2.2. Semi-Selective Strategy for Synthesis KIIIA with the Orthogonal Thiol Trt/Trt/Acm

**Figure 4:**
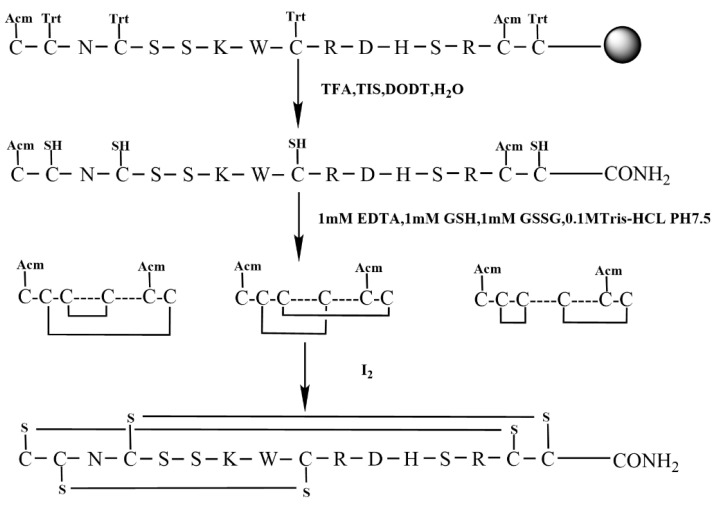
Semi-selective strategy for synthesis KIIIA (Scheme 2).

We employed the semi-selective strategy for the synthesis of KIIIA using S-Trt in combination with an S-Acm protecting group by Fomc chemistry. Acm group was applied to Cys1, and Cys15 with the remaining cysteine residues all protected by Trt groups (Figure 4).

**Figure 5:**
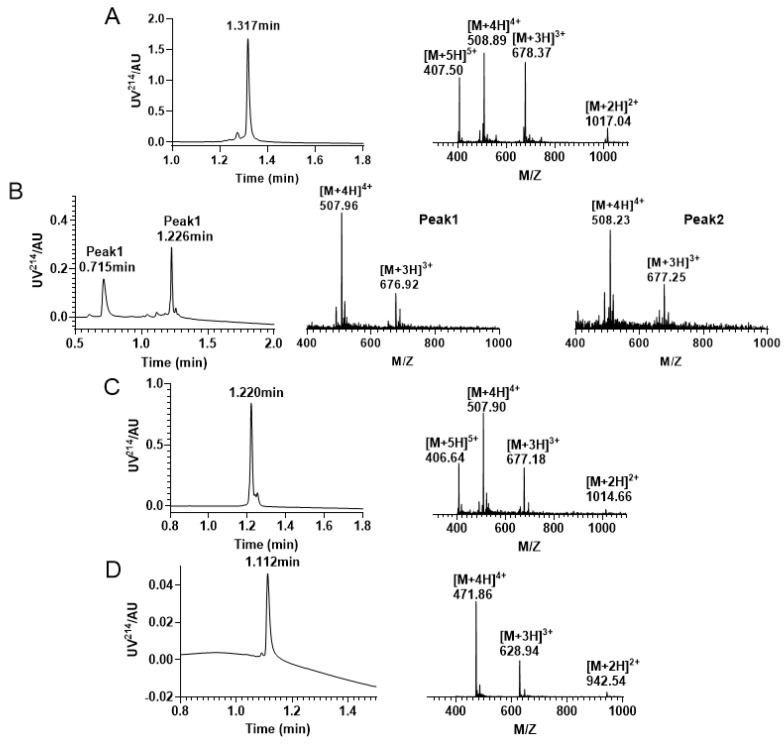
RP−UPLC and mass spectrogram monitoring of each step of reaction under Trt/Trt/Acm strategy folding. (**A**) RP−UPLC diagram of linear KIIIA with a retention time of 1.317 min and mass of 2032.11 Da; (**B**) A mixture of four pairs of free thiol free oxidation was observed. Peak 1 has a retention time of 0.715 min with a mass of 2027.76 Da; Peak 2 has a retention time of is 1.226 min with a mass of 2028.75 Da. (**C**) The RP−UPLC diagram of KIIIA with two disulfide bonds and two Acm-protecting groups, with a retention time of 1.220 min and a mass of 2028.54 Da. (**D**) RP−UPLC diagram of the purification after removal of the Acm protecting group to form the third pair of disulfide bonds, with a retention time of 1.112 min and a mass of 1883.82 Da.

In this study, we successfully synthesized the linear peptide KIIIA, as shown in Figure 5A. Subsequently, we performed oxidative folding of the peptide under the following conditions: 0.1 M Tris-HCI pH7.5 buffer, 1 mM EDTA, 1 mM reduced glutathione, and 1 mM oxidized glutathione. The first step of oxidation occurred in the four free thiol pairs Cys2, Cys4, Cys9, and Cys16, which theoretically produced three isomers. To monitor the process, we used RP−UPLC and analyzed successful disulfide bonding by mass spectrometry. Two peaks were identified to be generated during the first step of oxidative folding, as shown in Figure 5B. We then purified the oxidized folded isomer mixture by preparative RP−HPLC and obtained peak 2, as shown in Figure 5C. To confirm that we purified the second peak, we performed co-elution, as shown in Figure S3 (Supplementary Materials). Next, we removed the Acm-protecting group by iodine oxidation, and a third pair of disulfide bonds was formed between Cys1 and Cys15, as shown in Figure 5D.

### 2.3. Oxidative Folding Using the Regioselective Strategy with Trt/Acm/Mob Protecting Groups of Cysteines

**Figure 6:**
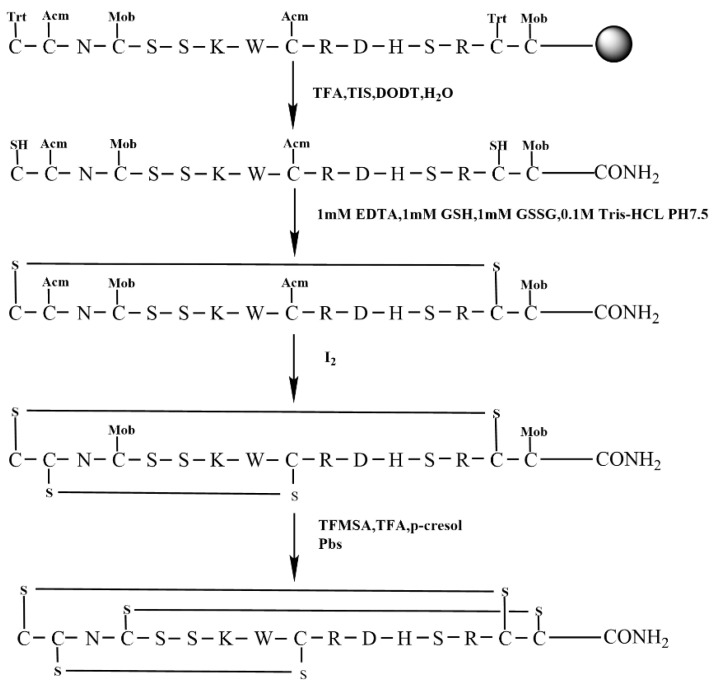
The scheme of regioselective oxidative folding of KIIIA with Trt/Acm/Mob orthogonal Cys protection (Scheme 3).

The regioselective strategy facilitates the selective generation of specific disulfide bonds, thereby guaranteeing the intended isomer. To facilitate this strategy, we synthesized the linear peptide KIIIA using the orthogonal Trt/Acm/Mob(4-methoxybenzyl) strategy. The Trt group protected the initial set of Cys residues, which are located in Cys1 and Cys15. The subsequent set of Cys residues, which were protected by Acm groups, are situated in Cys2 and Cys9. Finally, the third set of Cys residues, which were shielded by Mob groups, are positioned in Cys4 and Cys16, as shown in Figure 6. This strategic approach ensured the formation of the desired disulfide bonds and minimized the formation of unwanted byproducts (Figure 6).

**Figure 7:**
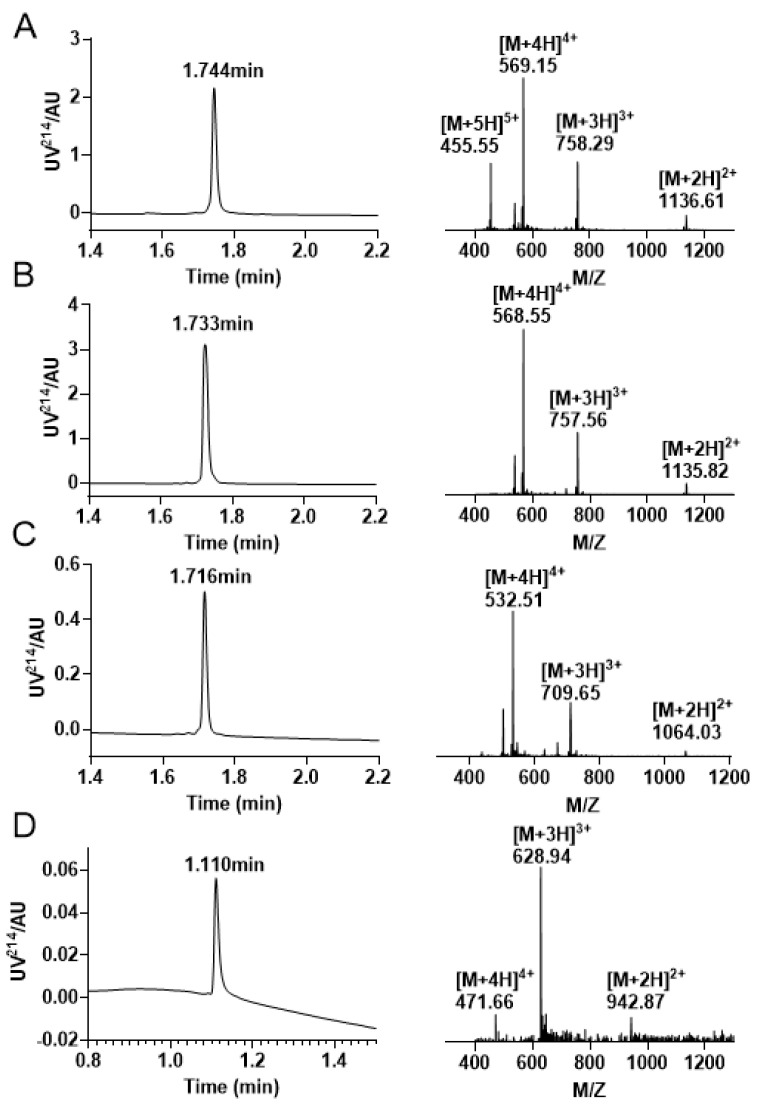
Analytical RP−UPLC chromatograms and ESI-MS Spectra of each stage of the Trt/Acm/Mob folding. (**A**) The linear peptide of KIIIA with a retention time of 1.744 min and a mass of 2271.87 Da. (**B**) The KIIIA has one disulfide bond between Cys1 and Cys15 with a retention time of 1.733 min and a mass of 2269.68 Da. (**C**) The RP−UPLC and ESI–MS profiles of KIIIA with Cys1 —Cys15/Cys2 —Cys9 linkage. The retention time is 1.716 min, and the mass is 2125.95 Da. (**D**) Chromatograms and mass spectra of the final oxidation results with a retention time of 1.110 min and a mass of 1883.82 Da.

Initially, the peptide was cleaved from the resin via the removal of side chain protecting groups, except for Cys(Acm) and Cys(Mob) (Figure 7A). Formation of the first disulfide bond between Cys1 —Cys15 using 0.1 M Tris −HCL PH7.5 buffer, 1 mM EDTA, 1 mM GSH, 1 mM GSSG. The first step of oxidative folding is between the free thiols. The product of the first step of oxidative folding was monitored by RP−UPLC and ESI−MS (Figure 7B).

The second stage of oxidative folding was executed in an iodine solution, which facilitated the removal of acetamidomethyl (Acm) and the formation of a second pair of disulfide bonds linking Cys2 and Cys9. Given that the concentration of iodine employed in the Acm removal process outlined in Scheme 2 was too severe for the stepwise oxidation approach, we carried out optimization of both the reaction time and concentration. The peptide was dissolved in an iodine solution at a concentration of 0.5 mg/mL, and the resulting product was subjected to purification by preparative RP−HPLC. The main peak was also identified using RP−UPLC and ESI−MS (Figure 7C).

Among all steps involved, the most arduous is the removal of the Mob group located on the third pair of Cys residues. Given that the elimination of Mob must not impact the two preexisting pairs of disulfide bonds, we opted to employ TFMSA/TFA/p-cresol for this purpose. Although the addition of TFMSA triggers a highly exothermic reaction leading to elevated temperatures, the reaction was carried out in an ice bath to prevent the formation of unpredictable by-products. Moreover, cold ether was added to the solution at the end of the reaction, following which the precipitate was obtained via centrifugation. However, the yield of this step was notably low. After the successful excision of Mob, a PBS solution was added to facilitate the formation of the final pair of disulfide bonds between Cys4 and Cys16, thereby completing the last stage of oxidative folding and obtaining the final product (Figure 7D).

### 2.4. Oxidative Folding Using the Regioselective Strategy with Acm/Mob/Trt Protecting Groups of Cysteines

**Figure 8:**
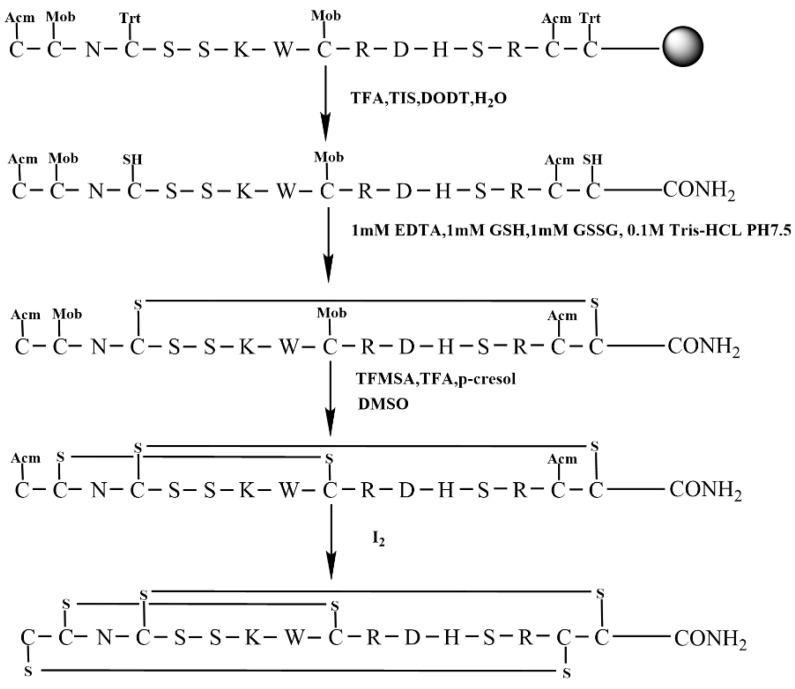
The scheme of regioselective oxidative folding of KIIIA with Acm/Mob/Trt orthogonal Cys protection. (Scheme 4).

This study aimed to examine the impact of different cysteine protection orders on KIIIA synthesis. We utilized Trt-protected Cys groups for Cys4 and Cys16 in the first pair of Cys residues, Mob-protected Cys groups for Cys2 and Cys9 in the second pair of Cys residues, and Acm-protected Cys groups for Cys1 and Cys15 in the third pair of Cys residues. Additionally, we modified the sequence of oxidation of the protecting groups during the formation of the disulfide bond in the last two steps. This process is illustrated in Figure 8.

**Figure 9:**
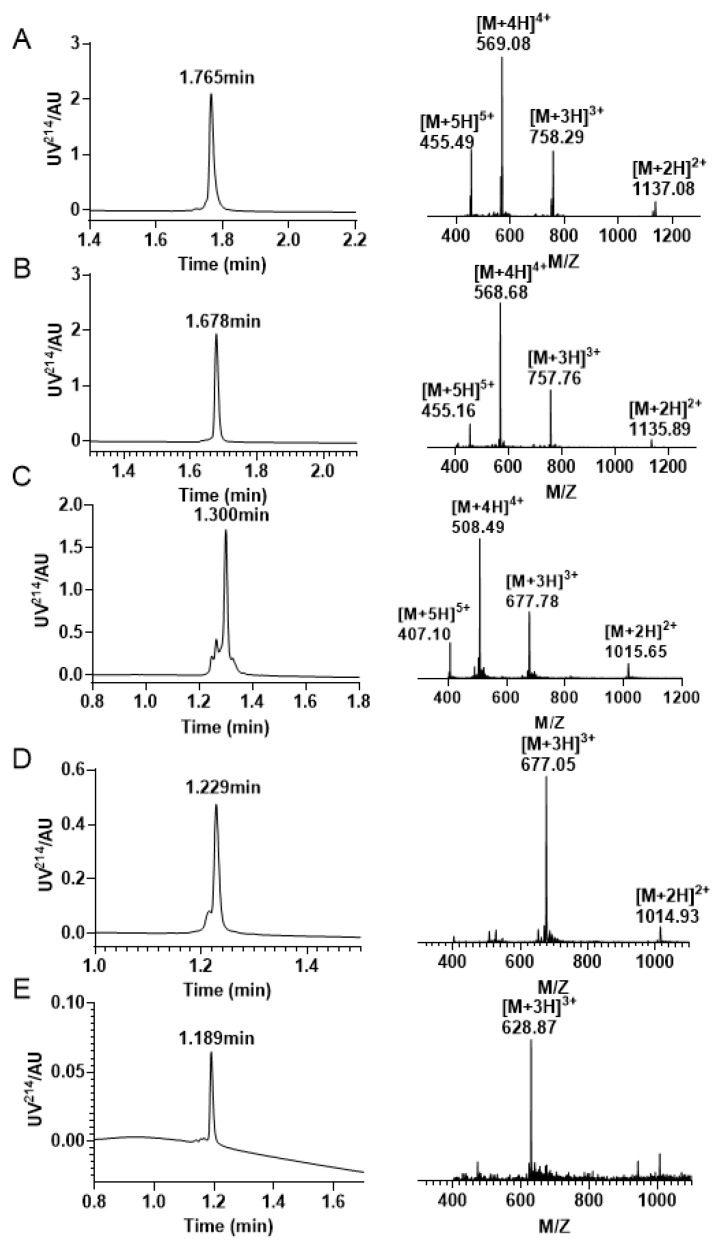
RP−UPLC and mass spectrogram were monitored at every step of the reaction during the Acm/Mob/Trt strategy folding. (**A**) The RP−UPLC and ESI−MS diagram after resin purification, with a retention time of 1.765 min and a mass of 2271.87 Da. (**B**) The RP−UPLC and ESI−MS diagram after the initial disulfide bond formation between Cys4 —Cys16, with a retention time of 1.678 min and a mass of 2270.28 Da. (**C**) The RP −UPLC diagram and ESI-MS diagram of the purified product after Mob protecting group removal, with a retention time of 1.300 min and a mass of 2030.34 Da. (**D**) The RP−UPLC diagram and ESI-MS diagram after the second disulfide bond is formed between Cys2 —Cys9, with a retention time of 1.229 min and a mass of 2028.15 Da. (**E**) The RP−UPLC diagram and ESI−MS diagram of the third pair of disulfide bonds formed by Cys1 —Cys15, with a retention time of 1.189 min and a mass of 1883.61 Da.

Similarly, we initially synthesized a linear peptide with distinct protecting groups and utilized 0.1 M Tris-HCl, 1 mM GSH/GSSG, and 1 mM EDTA to form a pair of disulfide bond between Cys4—Cys16 (Figure 9A,B). Following this, the Mob was removed, and we purified the peptide with two free thiols using preparative RP−HPLC (Figure 9C). Subsequently, DMSO was incorporated to create the second pair of disulfide bonds, and the resulting product was purified and isolated (Figure 9D). This strategy was employed to facilitate the formation of the second pair of disulfide bonds between Cys2 and Cys9. The ultimate step of disulfide bond linkage involves the elimination of Acm protective groups, which is the same as above strategy. The third pair of disulfide bonds in the Acm/Mob/Trt strategy is created between Cys1 and Cys15, and the final product was purified by RP−HPLC (Figure 9E).

### 2.5. Co-Elution Result of Oxidation Folding End Product

**Figure 10:**
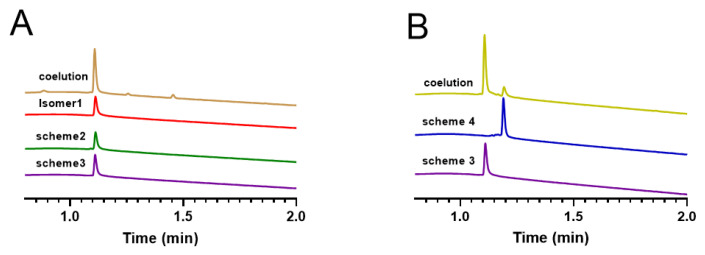
RP−UPLC analysis of oxidation products obtained by different synthetic strategies and products obtained by changing the position of the protecting group. (**A**) Isomer1 (red) coeluted with products of scheme 2 (green) and products of scheme 3 (purple). (**B**) The synthesized isomers of scheme 3 and scheme 4.

Here, we compared the oxidized folded products obtained from three different synthetic strategies, namely isomer 1 with Cys1 —Cys5, Cys2—Cys4, and Cys3—Cys6 linkage patterns, obtained by free oxidative isolation, the oxidized product of Scheme 2, and the oxidized product of Scheme 3. We analyzed the retention times of these products on RP−UPLC, and the results indicated that the retention times of all three products were consistent (Figure 10A). This demonstrated that the products of Scheme 2 were linked as Cys1 —Cys5, Cys2—Cys4, and Cys3—Cys6, and also verified the success of obtaining the same target products using different synthetic strategies. Subsequently, we compared Scheme 3 and Scheme 4, both of which were synthesized using a regioselective approach, with the only difference being the order of the protecting group and oxidation steps. We analyzed their retention times on RP−UPLC (Figure 10B) and found that although they have the same molecular weight, their retention times were different, indicating possible structural differences between the two schemes. To identify the step from which the two schemes differed, we eluted their linear peptide, the first step of oxidative folding product, and the second oxidative folding product again (Figures S4–S6), and found that they differed from the linear peptide onwards.

**Figure 11:**
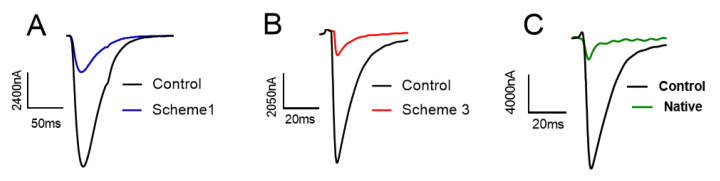
Current diagrams of KIIIA synthesis products using Scheme 1 and Scheme 3 (**A**,**B**) and their effects on blocking sodium channels at 1 µM concentration compared to natural isomers as controls (**C**).

We also used a regioselective approach to synthesize the natural isomers of KIIIA (Figure S1). The inhibitory activities of non-native KIIIA (the product of Scheme 1 and 3) and native KIIIA on NaV1.4 were evaluated using automated whole-cell membrane clamp electrophysiology in this study (Figure 11). The findings indicated that the isomers from Scheme 1 and Scheme 3 exhibited a blocking rate of 77.43% and 77.24%, respectively, on NaV1.4, while native KIIIA showed a blocking rate of 75.94%. These outcomes suggest that all synthesized KIIIA isomers have similar potency at a concentration of 1 µM, which is consistent with previous studies.

## 3. Discussion

The unique feature of cysteine residues is their ability to form reversible, covalent intramolecular, and intermolecular disulfide bonds through their thiol side chains. Disulfide bonds are also one of the most common post-translational modifications that occur during oxidative folding. The formation of disulfide bonds is crucial for stabilizing the entire three-dimensional arrangement of polypeptides, and it plays a vital role in biological function [34]. Similarly, the cysteine frameworks found in the conotoxin superfamilies are highly conserved and serve a critical function in forming disulfide bonds and stabilizing structures that are composed of a wide range of secondary structures, which are essential for receptor recognition, potency, and selectivity [3]. Thus, the precise regulation of disulfide bond formation is a crucial aspect of the chemical synthesis of conotoxins.

In general, the most active isomers of conotoxins are those found in their natural conformation, but there are exceptions, such as conotoxins AuIB and KIIIA [30,35]. The non-native disulfide bond isomers KIIIA, where it was shown that the unnatural Cys1—Cys9, Cys2—Cys15, and Cys3—Cys16 isomer was more potent than the native KIIIA [30]. In this study, we employed four distinct approaches to produce the most potent isomers of conotoxin KIIIA. One of the methods we utilized was free oxidation, which has been extensively utilized in the synthesis of various disulfide-bonded conotoxins, which also has been applied preliminarily in several studies for the synthesis of KIIIA [30,32,33,36]. This method offers several benefits, such as its straightforwardness, minimal synthetic purification steps, and relatively high yield. The drawback of this method is evident—multiple isomers may be generated, and additional identification is required to ensure correct disulfide bonding. By utilizing a regioselective approach, it is possible to achieve the directed formation of individual disulfide bonds, ensuring the desired isomer is produced. The two-step method offers a compromise between the one-step and three-step methods. During the first step of oxidative folding in this approach, two pairs of disulfide bonds are formed between the four free thiols, which theoretically results in three isomers. The final pair of disulfide bonds is generated by removing the Acm-protecting group with iodine oxidation [37]. In contrast, the regioselective oxidative folding strategy involves more intricate and time-consuming steps, resulting in low yields for three pairs of disulfide-bonded peptides.

The most significant finding of this study is that the targeted oxidation of the same triple disulfide bond resulted in isomers with identical molecular weights but varying retention times, which differs from previous research. As previously assumed, all prior studies suggested that the directed oxidation of the triple disulfide bond could generate the intended isomer [38,39,40]. However, this study reveals that the sequence and removal of the protective groups, as well as the order of disulfide bond oxidation, may affect the final structure of conotoxins. Schemes 3 and 4 yielded different isomers, presumably due to possible rearrangement of the disulfide bond during the removal of the Acm group by iodine oxidation. This study has a limitation in that we did not determine their 3D structures and explained the molecular-level differences between them. Moving forward, we aim to optimize the synthesis method and investigate the impact of cysteine protection sequence and cleavage order on the synthesis and structure of disulfide-bond-rich peptides.

In Fmoc chemistry, which is commonly used to synthesize disulfide-rich peptides, various combinations of protective groups, including trityl (Trt), acetamidomethyl (Acm), 4-methybenzyl (Meb)/4-methoxybenzyl (Mob), tert-butyl (tBu), and tert-butylthio (StBu), were used. Further research is necessary to determine how to select the most appropriate protective groups and cleavage sequence.

## 4. Materials and Methods

### 4.1. Linear Peptide Synthesis

To synthesize all linear peptides, we utilized standard solid-phase 9-fluorenylmethyloxycarbonyl (Fmoc) chemistry on a Liberty Blue automated peptide synthesizer. We used Rink Amide MBHA resin as the solid support and chose cysteine residues to protect them using triphenylmethyl (Trt), acetaminomethyl (Acm), and p-methoxybenzyl (Mob) approaches during the synthesis process. Coupling activation was achieved through DIC and Oxyma solutions, while Fmoc deprotection was performed using 20% piperidine in DMF.

We cleaved the resin using a solution of 92.5% TFA/2.5% triisopropylsilane/2.5% H_2_O/2.5% DODT and conducted the reaction for 30 min at 40 °C. This step simultaneously removed the side chain protecting groups, except for cysteines with Acm or Mob. After filtration of the cleavage solution, we added ice-cold ether to precipitate the peptides. The crude peptide was then centrifuged at 9000× *g* for 3 × 15 min, washed with ether, and dried to obtain the crude peptide.

### 4.2. Oxidative Folding with Random Free Oxidation

In the oxidation folding of Scheme 1, linear KIIIA was folded in a mixture containing 0.1 M Tris-HCl pH 7.5, 1 mM reduced glutathione, 1 mM oxidized glutathione, and 1 mM EDTA. The final peptide concentration in the folding mixture was 20 μM. After 2 h, the reaction was quenched by acidification with formic acid to a final concentration of 8%. The purification was performed on a Waters C18 column (250 × 19 mm, particle size 5 μm) using a preparative HPLC system (Waters Prep 150 LC system-2545). Solvent A was 0.1% (*v*/*v*) TFA in an aqueous solution, and solvent B was 60% (*v*/*v*) acetonitrile in an aqueous solution. A linear gradient of 2% to 90% of solvent B was used with a flow rate of 12 mL/min and an elution time of 45 min. The retention times of oxidative folding products were determined using the ACQUITY UPLC H-Class PLUS system. An ACQUITY UPLC BEH C18 column (Waters; 1.7 μm, 2.1 × 50 mm) was used, with a flow rate of 0.5 mL/min. The mobile phase B was varied from 5% to 80% within 3 min. Liquid phase tests were performed using both solvent A (0.05% TFA in H_2_O) and solvent B (90% ACN in H_2_O). Mass analysis was performed using Xevo TQD Triple Quadrupole Mass Spectrometry. The methods used in the following synthesis schemes for preparing the liquid phase, analyzing the liquid phase, and identifying using mass spectrometry are consistent with this.

### 4.3. Semi-Regioselective Strategy with Trt/Acm Protecting Groups of Cysteines

In the oxidative folding process of Scheme 2, the first two pairs of disulfide bonds were formed between Cys2, Cys4, Cys9, and Cys16 in a mixture containing 1 mM EDTA, 1 mM reduced glutathione, 1 mM oxidized glutathione and 0.1 M Tris-HCl at pH 7.5. The final concentration of the peptide in the folding mixture was 20 μM. After 2 h, the reaction was quenched by adding formic acid to a final concentration of 8%. The last pair of disulfide bonds between Cys1 and Cys15 was formed by dropping the peptide solution into an equal volume of 10 mM iodine dissolved in a mixture of ACN/H_2_O/TFA (24:71:5, *v*/*v*), oxidizing for 5 min, and terminating the reaction by adding VC. All products were analyzed by RP−HPLC and ESI-MS.

### 4.4. Regioselective Scheme 3 with Trt/Acm/Mob Protecting Groups of Cysteines

The peptide underwent a three-step oxidation process. The cysteine residues were protected by Trt, Acm, and Mob groups, respectively (Figure 6). In the first step, the Trt groups formed a disulfide bond through free oxidation, as described in Section 4.2. Subsequently, the Acm groups were removed by iodine oxidation in a 0.1% TFA/50% MeCN/H_2_O solution at a concentration of 0.5 mg/mL. Finally, the Mob protecting group was removed from the cysteine residue by dissolving the product in a solution containing p-cresol and TFA and cooling it to 0 °C. TFMSA (TFMSA/TFA/p-cresol 1:8:1) was added, and the mixture was stirred for 10 min at 0 °C. The resulting ether was poured into the lysate, and the precipitate was centrifuged at 9000 g for two sets of 15 min each. The resulting product was then sealed dry and purified using a preparative RP−HPLC. The last disulfide bond between Cys1 and Cys15 was closed with PBS oxidation for 30 min, followed by purification. The native isomer of KIIIA was synthesized using this method.

### 4.5. Regioselective Strategy with Acm/Mob/Trt Protecting Groups of Cysteines

The synthesis of scheme 4 was carried out according to the procedure shown in Figure 8. The first pair of disulfide bond was formed using free oxidation, as described in Section 4.2. Next, the Mob group was removed, and the disulfide bond between Cys2 and Cys9 was closed using DMSO oxidation for 4 h. The final disulfide bond between Cys1 and Cys15 was attached using the same method as described in Section 4.4. To this solution, 8 equivalents of MeCN were added, and the mixture was stirred for 20 min at room temperature. The excess I_2_ was reduced by adding ascorbic acid until the solution became colorless, and the Acm group was removed. All products of synthesis were identified using RP −UPLC and ESI-MS.

### 4.6. Electrophysiological Measurements

For RNA preparation, rat Nav1.4 (GenBank accession no. NM_013178) clones were obtained and transcribed with T7 after linearization with NotI. Oocytes were prepared by injecting 30 to 50 nL of cRNA in distilled water for sodium channel rNaV1.4 isoforms and incubating them in ND96 supplemented with antibiotics. Two-electrode voltage-clamp recording of sodium currents from oocytes was conducted with an OC-725C amplifier and a holding potential of −80 mV. Toxins were applied to oocytes by adding 3 μL of the peptide at 10 times its final concentration and measuring the time course of the block and recovery from the block.

## 5. Conclusions

This study addresses the challenging problem of synthesizing and oxidatively folding the disulfide-rich peptide KIIIA (C1 −C9, C2 −C15, C4 −C16). Different synthetic schemes were employed to synthesize peptides with the same target isomer, and it was found that altering the position of the protecting groups between the Trt/Acm/Mob strategy and the Acm/Mob/Trt strategy had a significant impact on the final product. These findings contribute to our understanding of the factors that influence the synthesis and folding of disulfide-rich peptides and may inform the development of more efficient and effective synthetic strategies in the future.

## Data Availability

The data presented in this study are available on request from the corresponding author.

## Supplementary Materials

The following supporting information can be downloaded at https://www.mdpi.com/article/10.3390/molecules28083377/s1. Figure S1: The scheme of regioselective oxidative folding of Native KIIIA with Trt/Acm/Mob or-thogonal Cys protection. Figure S2. Analytical RP−UPLC chromatograms and ESI-MS Spectra of each stage of the Trt/Acm/Mob folding. (A) The linear peptide of Native KIIIA with a mass of 2271.42 Da. (B) The Native KIIIA has one disulfide bond between Cys1 and Cys9 with a mass of 2269.53 Da. (C) The RP−UPLC and ESI−MS profiles of Native KIIIA with Cys1—Cys9/Cys2—Cys15 linkage. The mass is 2125.71 Da. (D) Chromatograms and mass spectra of the final oxidation results with a mass of 1883.61 Da. Figure S3. (A) Before purification of the oxidatively folded isomeric mixture in the first step of Scheme 2. (B)The main isomer obtained by the oxidative folding separation in the first step of Scheme 2 (C) Elution (red) of the main isomer (purple) obtained from the oxidative folding separation in the first step of Scheme 2 with the mixture of the unpurified isomers (blue). Figure S4. Coelution (green) of linear peptides in Scheme 3 (blue) and Scheme 4 (red). Figure S5. Coelution (blue) of oxidation folding products in the first step of Scheme 3 (green) and Scheme 4 (red) Figure S6. Coelution (blue) of oxi-dation folding products in the second step of Scheme 3 (green) and Scheme 4 (red).
