# Supplementary material for: Synthesis of the Most Potent Isomer of μ-Conotoxin KIIIA Using Different Strategies
Source: Molecules. 2023 Apr 11;28(8):3377. doi: 10.3390/molecules28083377 (PMC10143212; doi:10.3390/molecules28083377)
Supplement: Supplementary file 1 [file molecules-28-03377-s001.zip › molecules-2314712-supplementary.pdf]

Supplementary Material

Synthesis of the Most Potent Isomer of μ-Conotoxin KIIIA Using Different Strategies

Xunxun Jian [1], Yong Wu [1,*], Zaoli Mei [1], Xiaopeng Zhu [1], Dongting Zhangsun [1,2] and Sulan Luo [1,2,*]

1 School of Medicine, Guangxi University, Nanning 530004, China; janegut@163.com (X.J.); meizl1331@163.com (Z.M.); zhuxiaopeng@gxu.edu.cn (X.Z.); zhangsundt@163.com (D.Z.)
2 Key Laboratory of Tropical Biological Resources of Ministry of Education, Hainan University, Haikou 570228, China
* Correspondence: wuyong@gxu.edu.cn (Y.W.); sulan2021@gxu.edu.cn (S.L.)

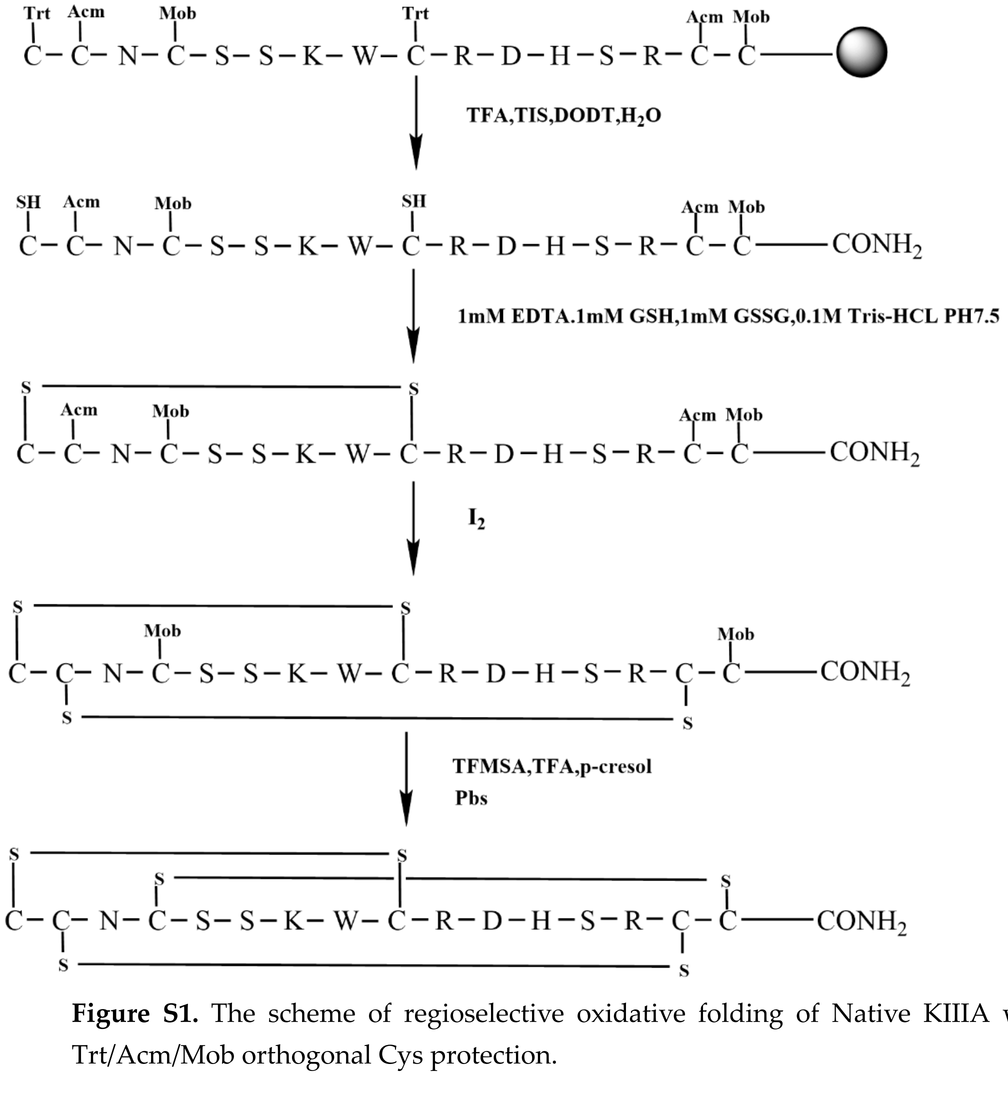

Figure S1. The scheme of regioselective oxidative folding of Native KIIIA with Trt/Acm/Mob orthogonal Cys protection.

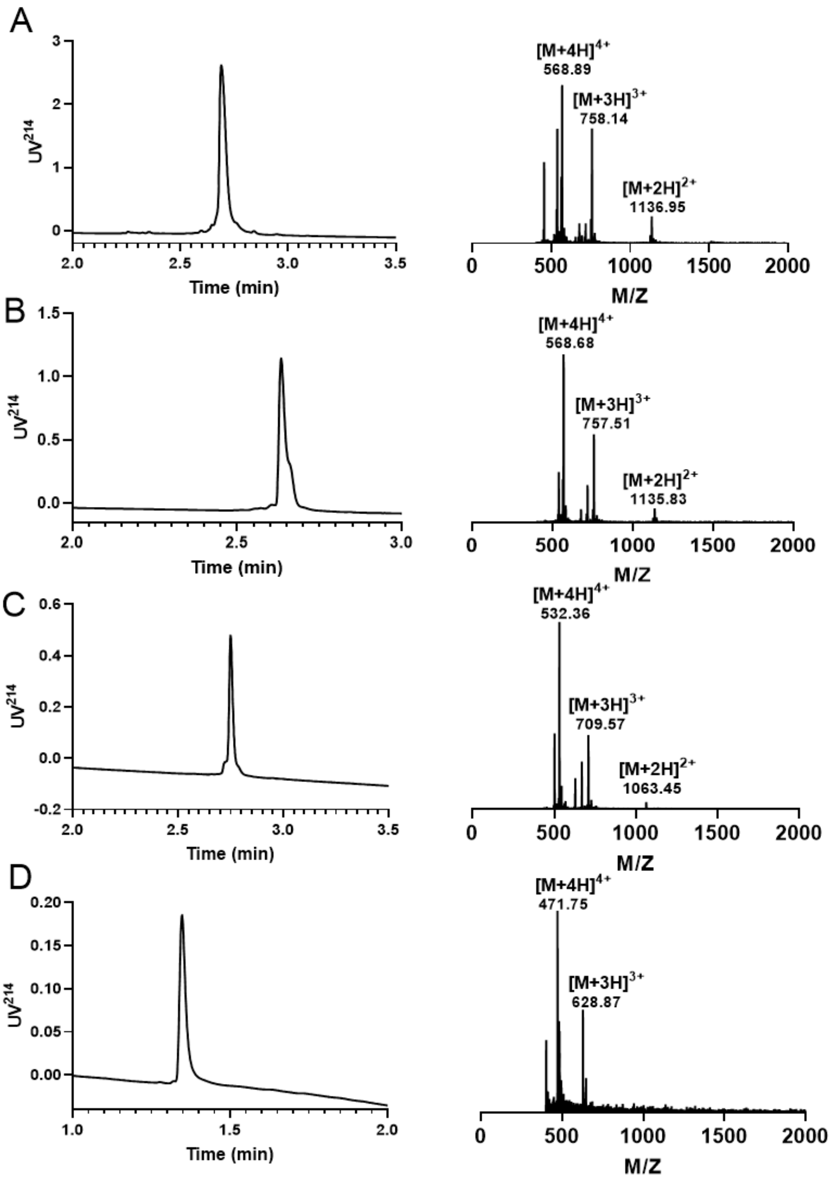

Figure S2. Analytical RP–UPLC chromatograms and ESI-MS Spectra of each stage of the Trt/Acm/Mob folding. (A) The linear peptide of Native KIIIA with a mass of 2271.42 Da. (B) The Native KIIIA has one disulfide bond between Cys^1 and Cys^9 with a mass of 2269.53 Da. (C) The RP–UPLC and ESI–MS profiles of Native KIIIA with Cys^1—Cys^9 /Cys^2—Cys^{15} linkage. The mass is 2125.71 Da. (D) Chromatograms and mass spectra of the final oxidation results with a mass of 1883.61 Da.

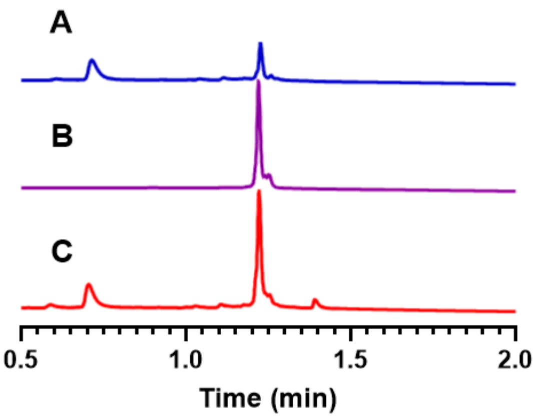

Figure S3. (A) Before purification of the oxidatively folded isomeric mixture in the first step of Scheme 2.(B)The main isomer obtained by the oxidative folding separation in the first step of Scheme 2 (C) Elution (red)of the main isomer (purple)obtained from the oxidative folding separation in the first step of Scheme 2 with the mixture of the unpurified isomers(blue)

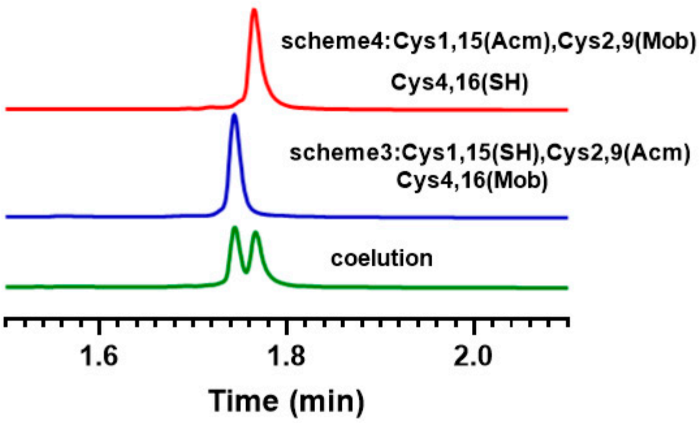

Figure S4. Coelution(green)of linear peptides in Scheme 3(blue) and Scheme 4(red)

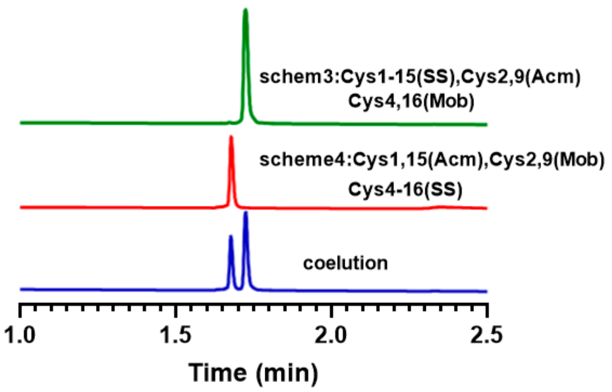

Figure S5. Coelution（blue）of oxidation folding products in the first step of Scheme 3(green)and Scheme 4(red)

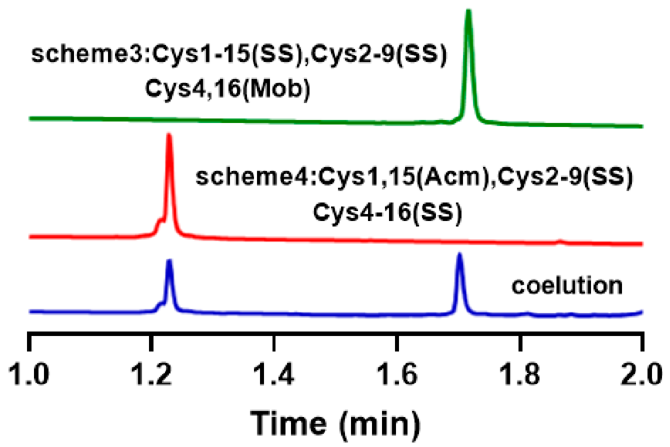

Figure S6. Coelution(blue)of oxidation folding products in the second step of Scheme 3 (green)and Scheme 4 (red)
